# Error Probability Mitigation in Quantum Reading Using Classical Codes

**DOI:** 10.3390/e24010005

**Published:** 2021-12-21

**Authors:** Francisco Revson Fernandes Pereira, Stefano Mancini

**Affiliations:** 1 School of Science and Technology, University of Camerino, I-62032 Camerino, Italy; stefano.mancini@unicam.it; 2 INFN, Sezione di Perugia, I-06123 Perugia, Italy

**Keywords:** quantum reading, cyclic codes, Reed–Muller codes, heterodyne receiver, Dolinar receiver

## Abstract

A general framework describing the statistical discrimination of an ensemble of quantum channels is given by the name quantum reading. Several tools can be applied in quantum reading to reduce the error probability in distinguishing the ensemble of channels. Classical and quantum codes can be envisioned for this goal. The aim of this paper is to present a simple but fruitful protocol for this task using classical error-correcting codes. Three families of codes are considered: Reed–Solomon codes, BCH codes, and Reed–Muller codes. In conjunction with the use of codes, we also analyze the role of the receiver. In particular, heterodyne and Dolinar receivers are taken into consideration. The encoding and measurement schemes are connected by the probing step. As probes, we consider coherent states. In such a simple manner, interesting results are obtained. As we show, there is a threshold below which using codes surpass optimal and sophisticated schemes for any fixed rate and code. BCH codes in conjunction with Dolinar receiver turn out to be the optimal strategy for error mitigation in quantum reading.

## 1. Introduction

Quantum state discrimination composes an important part of several quantum computing protocols [1]. Quantum communication relies upon the ability of the receiver to distinguish between a set of information carriers [2,3]. The security of quantum key distribution protocols is based on the impossibility of perfectly distinguishing non-orthogonal states [1]. In both situations, where one needs to show that states are distinguishable or not, the set of quantum states are fixed and an analysis over the minimum achievable error probability is performed. In order to decrease the error probability, the only possible method is optimizing the measurement apparatus. There are paradigms giving more freedom and, therefore, increasing the complexity for analyzing them.

A natural extension of the task of discriminating quantum states is envisioned in quantum channel discrimination [4,5,6,7,8,9,10,11,12,13], and, more generally, quantum reading [14]. Quantum reading task is important for modeling and quantifying quantum memories as a useful resource. Information-theoretically bounds, communications protocols, and error probability results can be applied to quantum reading. Quantum reading considers the use of input and output quantum resources to enhance the retrieval of classical information stored in quantum channels. Actually, it considers that one can record bits of information in memory cells by storing a quantum channel picked from a given ensemble. The goal in quantum reading is to optimize the probing strategy as well as the encoding and decoding protocols to reduce the error probability in the discrimination process.

Efficient paths to quantum reading can be foreseen by the use of coding techniques [15,16,17]. On the first hand, quantum error-correcting codes give interesting candidates to probe the memory cells. The structure of their Hilbert subspace makes them reliable to some dissipator noise due to system-environment interaction [18]. On the other hand, the encoding process can be implemented using classical error-correcting codes in order to add redundancy and increase error mitigation. Focusing on error probability mitigation as the figure of merit and using short-length classical codes, we consider three families: Reed–Solomon codes, BCH codes, and Reed–Muller codes [19]. These families have a large diversity of parameters, and their values can be easily controllable. Additionally, the existing low-complexity encoding and decoding algorithms make the protocol proposed in this paper realizable with the current technology.

Following the previous reasoning, we consider a quantum reading task where the ensemble is composed of two quantum channels. Each quantum channel is modeled by a pure-lossy bosonic channel with different transmissivities. For the analysis, we will consider the three families of codes mentioned before, two types of receivers, and a probing state. The first receiver is a heterodyne receiver [2]. The use of heterodyne receiver is justifiable due to the phase-insensitivity property of pure-lossy channels. The second one is a Dolinar receiver [2]. This is an adaptive receiver that can achieve the Helstrom bound on distinguishing two quantum states [2]. There is an intrinsic complexity in implementing this receiver, mostly due to the adaptive and fast response characteristics. However, it has been implemented in practice, where its optimality was shown [20]. Lastly, for the probing state, we consider coherent states. Therefore, we aim to show that the improvements and results obtained in the proposed scheme is due to the classical codes and receivers considered.

For any of the three families of classical error-correcting codes considered, we show improvements when compared with optimal strategies using coherent or squeezed states. We compute the average number of photons needed to surpass the optimal strategies for fixed code, rate, and receiver. This value defines a threshold. There are strategies giving a lower threshold. Using the heterodyne receiver, the best strategy is the BCH codes. However, there are some values of rate where Reed–Muller codes have similar performance. Using the Dolinar receiver, BCH and Reed–Solomon codes have almost the same performance. For lower values of rate, the BCH codes perform better than Reed–Solomon. The situation changes when the rate is above 0.45. For any of the codes, the best performances are obtained using the Dolinar receiver. We are able to achieve thresholds in this strategy for an average number of photons below 18. Furthermore, in order to achieve the threshold for a large range of rates, one does not need more than 6 photons per probing state.

This paper establishes an important basis for the use of classical codes in quantum reading. In particular, it has taken the novel path of proposing an explicit use of short-length classical codes and showing how much they impact error mitigation. These results demonstrate the possibility of improving the current technology used on quantum memories by low complexity encoding and decoding schemes. The values of the threshold obtained emphasize this statement. A relevant achievement of this paper is the explicit characterization of error mitigation in terms of different code lengths and code families. One can obviously expect error mitigation by increasing the number of photons used to probe the memory cells, but what can happen when associating classical codes to the scheme is answered quantitatively in this paper.

Due to the novel path taken in this paper by considering classical codes and the error mitigation produced by them, there is not much to be said about the connection with the existing literature. The only related work in the literature is Ref. [17], where the authors proposed polar coding and decoding schemes to achieve the reading capacity. However, the results in Ref. [17] can only be appreciated for large code lengths. One can also find unrelated approaches to quantum reading, such as Ref. [21], where it is shown that entanglement-assisted probing outperforms classical strategies on barcodes data, without any explicit analysis of encoding and decoding schemes.

This paper is organized as follows. In Section 2, we present the main concepts used throughout the paper. A description of the three families of classical codes is given. Additionally, we explain the task of quantum reading and the quantum channel model considered. Next, in Section 3, we describe the proposed protocol. It is divided into three parts: probing strategy, encoding scheme, and decoding scheme. The performance analysis of the protocol is given in Section 4. Lastly, we draw our conclusions in Section 5.

## 2. Preliminaries

This section introduces the main concepts of classical codes and quantum reading needed for this paper. We begin with a brief overview of cyclic codes and then specialize in Reed–Solomon and BCH codes. Subsequently, we show a construction method for Reed–Muller codes that is similar to Reed–Solomon codes. Lastly, the quantum reading task is introduced. The general concept is given, followed by a detailed description of the channel model adopted.

### 2.1. Classical Codes

#### 2.1.1. Cyclic Codes

A linear code *C* over $F_{q}$ with parameters ${\left[n,k,d\right]}_{q}$ is called cyclic if for any codeword $(c_{0},c_{1},\ldots ,c_{n-1})\in C$ implies $(c_{n-1},c_{0},c_{1},\ldots ,c_{n-2})\in C$. Defining a map from $F_{q}^{n}$ to $F_{q}\left[x\right]/\left(x^{n}-1\right)$, which takes $c=\left(c_{0},c_{1},\ldots ,c_{n-1}\right)\in F_{q}^{n}$ to $c\left(x\right)=c_{0}+c_{1}x+\cdots +c_{n-1}x^{n-1}\in F_{q}\left[x\right]/\left(x^{n}-1\right)$, we can see that a linear code *C* is cyclic if and only if it corresponds to an ideal of the ring $F_{q}\left[x\right]/\left(x^{n}-1\right)$. Since any ideal in $F_{q}\left[x\right]/\left(x^{n}-1\right)$ is principal, then any cyclic code *C* is generated by a polynomial g(x), which divides $\left(x^{n}-1\right)$. These polynomials are called generator polynomials.

A way to characterize the parameters of a cyclic code is by means of the generator polynomial and its defining set. Roughly speaking, the defining set characterizes the common zeros of all polynomials c(x)∈C. More precisely, let *n* and *q* be relative prime, so $q^{e}\equiv 1\;\mathrm{mod}\;n$, for some integer *e*. Fix an element β of order *n* in an extension $F_{q^{e}}$ of $F_{q}$. We have that the defining set of *C*, which is denoted by Z(C), is $Z\left(C\right)=\{i\in Z_{n}:c\left(\beta^{i}\right)=0\;\mathrm{for}\;\mathrm{all}\;c\left(x\right)\in C\}$. The family of Reed–Solomon (RS) codes is a particular case of cyclic codes, where the generator polynomial has some additional properties.

**Definition** **1.** *Let α be a primitive element of $F_{q}$. Let b≥0, n=q−1, and 1≤k≤n. A cyclic code $RS_{k}\left(n,b\right)$ of length n over $F_{q}$ is a* Reed–Solomon (RS) code *if the generator polynomial is given by*

$$g\left(x\right)=\left(x-\alpha^{b}\right)\left(x-\alpha^{b+1}\right)\cdot \cdots \cdot \left(x-\alpha^{b+n-k-1}\right).$$

Since the minimal distance of any cyclic code is bounded from below by the maximum number of consecutive elements in Z(C) and the Singleton bound says that the minimal distance of a [n,k] code is not greater than n−k+1, we see that RS codes have minimal distance equal to n−k+1. Thus, for fixed length and dimension, they have maximal possible minimal distance and, therefore, they are named maximal distance separable (MDS) codes.

Even though the previous definition of RS codes describes them properly, there is a more practical way to construct RS codes. Choose an enumeration $P=(P_{1},\ldots ,P_{n})$ of *n* mutually distinct points in $F_{q}$. Let $F_{q}\left[X\right]$ be the set of all polynomials in the variable *X* with coefficients in $F_{q}$. The RS code is given by

(1) $$RS_{k}\left(n,b\right)=\left\{ev_{P}\left(f\right):f\in F_{q}\left[X\right],\deg\left(f\right)<k\right\},$$

where ev is the evaluation map defined by

(2) $$\begin{array}{ccc} ev_{P}:F_{q}\left[X\right] & \to & F_{q}^{n}, \end{array}$$

(3) $$\begin{array}{ccc} f(X) & \mapsto & (f\left(P_{1}\right),\ldots ,f\left(P_{n}\right)). \end{array}$$

For the decoding scheme, we apply the Berlekamp–Massey algorithm [22] (Section 5.4.2). Since we are using small RS codes, meaning that the length of the RS codes is significantly shorter than LDPC or Turbo codes, the Berlekamp–Massey algorithmic fulfills our needs.

Before presenting BCH codes, we need to introduce the concept of minimal polynomials. Let $\beta \in F_{q^{e}}$. The minimal polynomial over $F_{q}$ of β is the monic polynomial with smallest degree, M(x), and coefficients in $F_{q}$ such that M(β)=0. If $\beta =\alpha^{i}$ for some primitive *n*th root of unity $\alpha \in F_{q^{e}}$, we denote the minimal polynomial of β by $M^{\left(i\right)}\left(x\right)$.

**Definition** **2.** *Let $F_{q}$ be a finite field, n and q be relative prime, and α be a primitive nth root of unity. A cyclic code BCH(δ,b) of length n over $F_{q}$ is a Bose–Chaudhuri–Hocquenghem (BCH) code of design distance δ if the generator polynomial is given by*

(4) $$g\left(x\right)=\mathrm{lcm}\{M^{\left(b\right)}\left(x\right),M^{\left(b+1\right)}\left(x\right),\ldots ,M^{\left(b+\delta -2\right)}\left(x\right)\},$$

*for some integer b≥0. If $n=q^{e}-1$ then the BCH code is called primitive and if b=1 it is called narrow-sense.*

The dimension of a BCH code is equal to k=n−deg(g(x)), similarly to the Reed–Solomon code case. The minimal distance can also be computed using the defining set of BCH(δ,b). However, there is no general formula for BCH codes. We would need to introduce the concept of *q*-ary cyclotomic coset modulo *n* and analyze each code in order to obtain the respective dimension. Therefore, it is beyond the scope of this paper. The performance analysis of the code is based on error probability and rate.

The encoding algorithm used for BCH codes is implemented via matrix multiplication, which has complexity $O(n^{2})$. For the decoding algorithm, the Berlekamp–Massey algorithm is also used.

#### 2.1.2. Reed–Muller Codes

The construction of Reed–Muller codes is similar to the one presented for Reed–Solomon codes using evaluation map. The difference relies on the set of polynomials considered. Take a vector space $F_{q}^{m}$, where *m* is an integer. Choose an enumeration $P=(P_{1},\ldots ,P_{n})$ of *n* mutually distinct points in $F_{q}^{m}$. The evaluation map is defined as

(5) $$\begin{array}{ccc} ev_{P}:F_{q}\left[X_{1},\ldots ,X_{m}\right] & \to & F_{q}^{n}, \end{array}$$

(6) $$\begin{array}{ccc} f(X_{1},\ldots ,X_{m}) & \mapsto & (f\left(P_{1}\right),\ldots ,f\left(P_{n}\right)), \end{array}$$

where $F_{q}\left[X_{1},\ldots ,X_{m}\right]$ is the set of all polynomials in the variables $X_{1},\ldots ,X_{m}$ with coefficients in $F_{q}$. We can define Reed–Muller codes by means of evaluating polynomials.

**Definition** **3.** *Let $F_{q}$ be a finite field, and r,m be integers such that 0≤r<m(q−1). Let $n=q^{m}$ and $P=(P_{1},\ldots ,P_{n})$ be an enumeration of all elements in $F_{q}^{m}$. A block code RM(r,m) of length n over $F_{q}$ is a Reed–Muller code of order or degree r in m variables if it is given by the set*

(7) $$RM\left(r,m\right)=\{ev_{P}\left(f\right):f\in F_{q}\left[X_{1},\ldots ,X_{m}\right],\deg\left(f\right)\leq r\}.$$

It is possible to show that the dimension of a RM(r,m) over $F_{q}$ is equal to the size of the set [19] (Proposition 5.4.7)

(8) $$\begin{array}{cc} E_{q}\left(r,m\right)=\{e\in N_{0}^{m}: & 0\leq e_{i}\leq q-1\;\mathrm{for}\;\mathrm{all}\;i\\ & \;\mathrm{and}\;e_{1}+\cdots +e_{m}\leq e\}. \end{array}$$

The encoding algorithm used to construct the Reed–Muller codes is via generator matrix. We use the standard majority logic vote method due to Irving S. Reed [23] in order to decode the received string.

### 2.2. Quantum Memory Cell and Quantum Reading

We now provide a description of quantum memory cells and quantum reading suitable for this paper. A quantum memory cell is defined as the set $\Phi ={\left\{W^{x},p_{x}\right\}}_{x\in X}$ of quantum channels. For a fixed *x*, quantum input and output Hilbert spaces $H_{B^{\prime }}$ and $H_{B}$, respectively, we have

(9) $$\begin{array}{cc} W^{x}:D\left(H_{B^{\prime }}\right) & \to D(H_{B}) \end{array}$$

(10) $$\begin{array}{cc} \rho & \mapsto W^{x}\left(\rho \right), \end{array}$$

where $D\left(H_{B^{\prime }}\right),D\left(H_{B}\right)$ are the sets of input and output density states of the quantum channel $W^{x}$, and $p_{x}=P_{X}\left\{X=x\right\}$ is the probability distribution law of *X*. We call x∈X the quantum memory cell index. We consider X binary and the distribution of the random variable *X* describing the label of the quantum channels to be Bernoulli with probability p=1/2.

**Figure 1:**
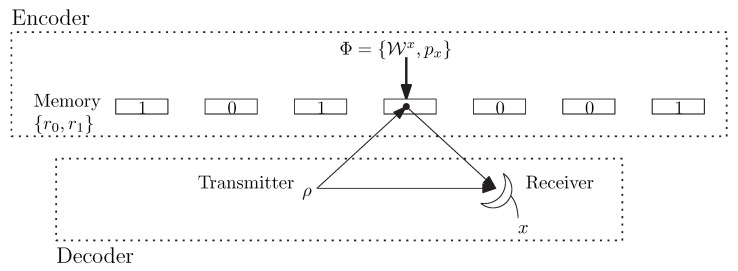
Quantum reading protocol.

The quantum reading protocol consists in probing a memory cell in order to discriminate between its possible index values, i.e., between quantum channels. Additionally, and more important to this paper, it is assumed that the encoder can use classical codes during the writing process on the quantum channels arising from the quantum memory cell in order to reduce the error probability. Let c be a codeword of a classical code. Then, the encoder is able to choose the quantum memory cell index and, therefore, which channel is to be placed, according to c. Since the encoder chooses the quantum memory cell index, we can assume, without loss of generality, that a source code is performed on the information bits to produce evenly distributed indexes. A schematic of the quantum reading protocol is given in Figure 1.

For the quantum channel model, we use bosonic pure-lossy channels. They are a reasonable basic continuous variables model for optical memories. Thus, the binary channel ensemble is given by $\Phi ={\left\{W^{x},p_{x}\right\}}_{x=0,1}$, where $p_{0}=p_{1}=1/2$, and $W^{x}$ represents a pure-lossy channel with transmissivity $0\leq \kappa_{x}\leq 1$. The action of each $W^{x}$ channel in the Heisenberg picture is described by the map

(11) $${\hat{a}}_{B}\to \sqrt{\kappa_{x}}{\hat{a}}_{B}-\sqrt{1-\kappa_{x}}{\hat{a}}_{E},$$

where ${\hat{a}}_{B}$ is the annihilation operator of the probe mode and ${\hat{a}}_{E}$ is that of an environmental vacuum mode. We are considering that the two parameters $\kappa_{0}$ and $\kappa_{1}$ assume values in the interval [0,1], since it is considered that the optical memories are read by reflection.

#### Optimal Error Probability

In Ref. [15], the error probability under the optimization of the probe state and the measurement apparatus used in the receiver is analyzed. The optimization considers that the decoder can probe the memory cells as many times as necessary, and Helstrom’s measurements are at its disposal. Using coherent states, they show that the optimal error probability is

(12) $$P_{c}=\frac{1-\sqrt{1-\exp[-\bar{n}\left(\sqrt{\kappa_{0}}-\sqrt{\kappa_{1}}\right)]}}{2},$$

where $\bar{n}$ is the average number of photons. On the other hand, they also consider non-classical probes described via Einstein–Podolsky–Rosen transmitter. It is composed of *s* pairs of signals and references, entangled via two-mode squeezing. The squeezing parameter ξ of the two-mode squeezing state and the parameter *s* are connected by the expression $\xi =\mathrm{arcsinh}\sqrt{\frac{\bar{n}}{s}}$. Optimizing the error probability in terms of the parameter *s*, the following expression of the error probability is obtained

(13) $$P_{s}=\frac{\exp(-\mu \bar{n})}{2},$$

where

(14) $$\mu =\frac{\kappa_{0}+\kappa_{1}+2}{2}-2\sqrt{\kappa_{0}\kappa_{1}}-\sqrt{\left(1-\kappa_{0}\right)\left(1-\kappa_{1}\right)}.$$

In the following section, we are going to analyze the error probability derived from the use of classical codes and compare it with the previous optimal error probabilities.

## 3. Proposed Protocol

This section is divided in two. Initially, we present the probing strategy applied to the memory cells. Afterward, the encoding and decoding protocols implemented to incorporate and retrieve information from the memory cells are shown. In particular, the heterodyne and Dolinar receivers used to measure the output probe state are described.

### 3.1. Probing Strategy

Choosing the probing state is an important step in any metrological system. However, our focus is on the improvement of using classical codes. Therefore, we have opted to apply single-mode coherent states in the probing step. Their implementation is not so complex as compared with squeezed states, and one can also use standard optical devices to manipulate the phase and amplitude of the coherent state.

We are going to describe a single-mode coherent state via the basis of Fock states and the Wigner function. The first characterization will help us to compute the probability distribution obtained in the photodetector. The second characterization is important when we consider the action of the displacement operator and the description of the probability distribution in a heterodyne detection.

Let {|n〉}, n=0,1,2,…, be the basis of Fock states. A single-mode coherent state |α〉 can be written as

(15) $$|\alpha \rangle =e^{\frac{1}{2}{\left|\alpha \right|}^{2}}\sum_{n=0}^{\infty }\frac{\alpha^{n}}{\sqrt{n!}}|n\rangle .$$

Its Wigner function reads

(16) $$W_{\alpha }\left(r\right)=\frac{2}{\pi }e^{-{\left(r-\bar{r}\right)}^{T}\left(r-\bar{r}\right)},$$

where

(17) r¯=2Re{α}2Im{α}.

Since the Wigner function gives a quasiprobability description of a quantum state, we see that a coherent state is Gaussian.

### 3.2. Encoding and Decoding Protocols

The encoding process consists in storing the information to be read in the future. Suppose we have an information vector i to be stored. Due to imperfect storing or reading of the information vector, the noise inherently prohibits us from reading the information perfectly. We need to add redundancies in order to overcome this issue and, in our case, use classical codes. Therefore, we first produce a codeword over $F_{2}$ written in the memory cell labels. Suppose the information vector is a *K* bits string, the encoder uses i to derive the codeword $c=(c_{1},\ldots ,c_{N})$ of length *N*. If the classical code is constructed over $F_{2^{s}}$, with s>1 an integer, then it is needed to choose a basis of $F_{2^{s}}$ over $F_{2}$ and represent each coordinate $c_{j}$, 1≤j≤N, in that basis. This basis expansion process will be implemented when we use Reed–Solomon codes. After producing the codeword c over $F_{2}$, we can move to the second step, which consists of associating each coordinate of c to the corresponding quantum memory cell index.

Observe that we are assuming that the quantum memory cell is composed of two quantum channels. However, the same method can be extended to quantum memory cells with cardinality $p^{s}$, where *p* is a prime number and s≥1 is an integer.

The decoding protocol consists of two parts. The first one is measuring the probing state in order to estimate the quantum memory cell. After probing the set of *N* memory cells, a noisy string or vector is obtained. Below we present in detail the measurement apparatus used through the paper, the heterodyne and Dolinar receiver. After producing the noisy vector, a decoding algorithm computes the best candidate of codeword for the classical code in consideration. The decoding algorithms used for the codes used in this paper have been discussed in Section 2.

#### 3.2.1. Heterodyne Receiver with Maximum Likelihood Estimator

Suppose we plan to recover the information stored in the memory cells, then we probe each memory cell with a coherent state. The output state contains the information stored in the memory cell. The lossy channel will give $\kappa_{x}\alpha$, for x=0,1, once applied to the coherent state α. So, we need to retrieve the information about *x*. For this goal, the heterodyne and Dolinar receiver are used. The heterodyne receiver is presented in this subsection, and Dolinar receiver in the following.

**Figure 2:**
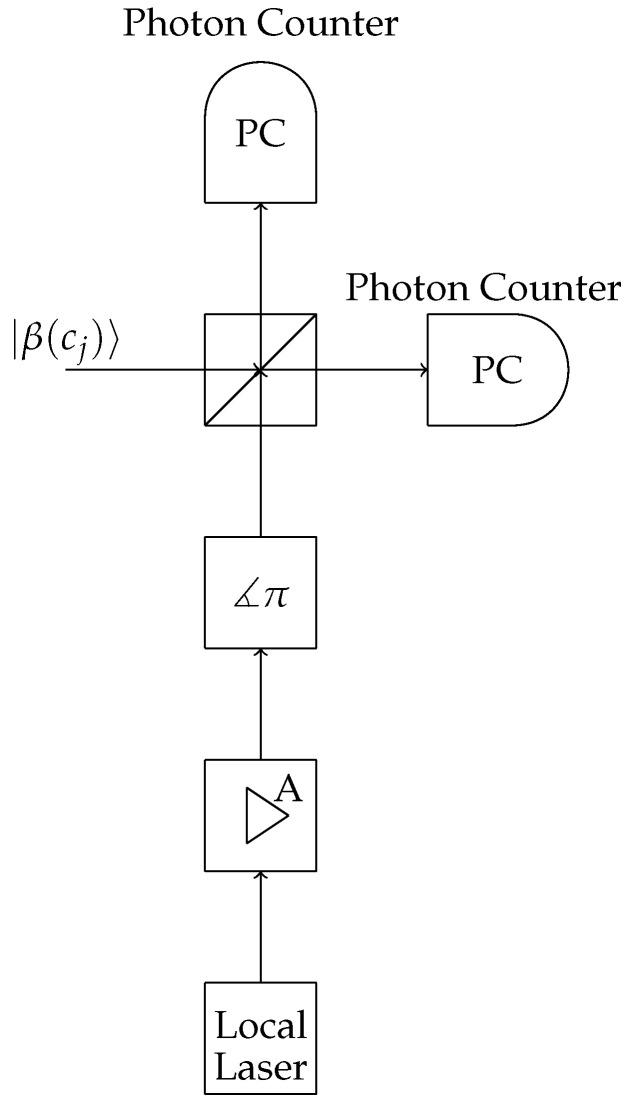
Heterodyne receiver schematic.

Heterodyne receivers are used when one wishes to retrieve the information stored in the parameter α of the coherent state [2]. See Figure 2. The POVM describing this receiver is

(18) $$\Pi_{\beta }=\frac{1}{\pi }|\beta \rangle \langle \beta |.$$

Thus, the probability distribution obtained after probing the memory cell in the *j*-th position and using a heterodyne receiver is given by

(19) $$p_{Y}\left(\beta \right)=\frac{1}{\pi }\beta |W^{c_{j}}\left(|\alpha \rangle \langle \alpha |\right)|\beta \rangle ,$$

where β∈C and *Y* is the random variable describing the output probability distribution. After this step, we need to divide the complex plane in order to estimate if $c_{j}$ is equal to zero or one. This is implemented via the maximum likelihood estimator. The partition of the complex plane and the decision rules are described by

(20) $${\hat{c}}_{j}=\left\{\begin{array}{cc} 1, & \mathrm{if}\;\Lambda (\beta )\geq \eta ,\\ 0, & \mathrm{if}\;\Lambda (\beta )<\eta , \end{array}\right.$$

where

(21) $$\Lambda \left(\beta \right)=\frac{p_{Y|X}\left(\beta |1\right)}{p_{Y|X}\left(\beta |0\right)}$$

and

(22) $$\eta =\frac{p_{0}}{p_{1}},$$

with $p_{0}$ as the probability of 0 in a codeword c and $p_{1}=1-p_{0}$. From the Wigner function of the coherent state, we see that the distribution of $p_{Y|X}\left(\beta |x\right)$, x=0,1, is Gaussian. Therefore, the partition of the plane can be made from the parameter of the coherent state and the reflexivities of the memory cells.

#### 3.2.2. Dolinar Receiver

**Figure 3:**
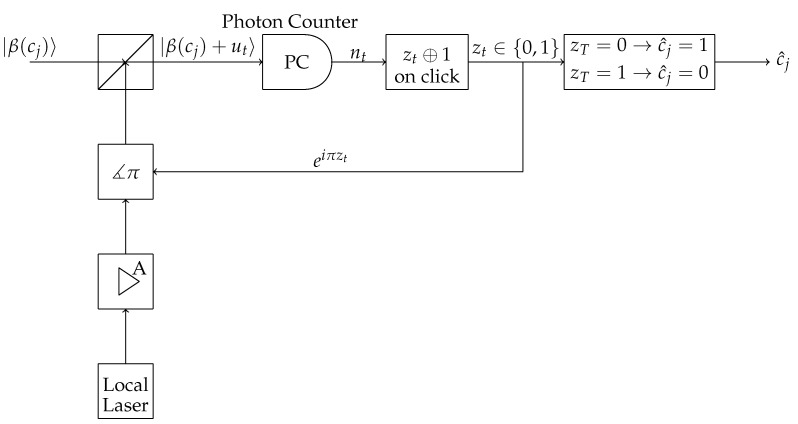
Dolinar receiver schematic.

Dolinar receiver takes a different road than the heterodyne receiver [2]. It is an adaptive receiver. Suppose there are two coherent states, |α〉 and |γ〉, we want to distinguish. Initially, the Dolinar receiver makes a guess of the true state and subtracts it from the state to be determined. This leads to the state |ψ−ϕ〉, where ψ is the state to be determined and ϕ is the guess. Next, the resulting state is measured in a photodetector. For the computational simulation, we use

(23) $$Q_{1}=\sum_{n=1}^{\infty }{\left(1-\eta \right)}^{n}|n\rangle \langle n|,$$

where 1−η is the detection efficiency, $Q_{1}$ is the click operator and $Q_{0}=I-Q_{1}$ is the no-click operator. If there is a click, then the guess is changed and the process is repeated with the same state to be determined. Otherwise, the receiver declares that the true state is the one it has guessed. An illustrative schematic is shown in Figure 3.

For numerical and analytical analysis of the above process, we need to clarify the feedback process shown. First of all, we are considering that the feedback is fast. Consider that the feedback runs *l* times and the incoming state is |α〉. Then, in each round, the receiver has $|\frac{\alpha }{\sqrt{l}}\rangle$ as the input state. This is the state that the receiver has to guess. Observe that increasing too much the number of rounds, *l*, in the receiver will make it more susceptible to errors. In particular, we will use l=2 in the following.

The second point is about the subtracted state |ϕ〉. To optimize the error probability, |ϕ〉 needs to be of time-varying amplitude as shown in [2]. For simplicity, we are assuming that |ϕ〉 can be instantaneously changed into one of the two possible states. Even though not optimal, this strategy gives significant improvements when compared with the heterodyne receiver. See the following section.

## 4. Analysis of Error Probability Using Classical Codes

Consider memory cells $\left\{W^{0},W^{1}\right\}$, where $p_{0}=p_{1}=1/2$, with reflexivities $\kappa_{0}$ and $\kappa_{2}$, respectively. Suppose a string of memory cells is encoded via a classical code C. The information stored in the string is retrieved by probing them via a coherent state. From the result obtained, we analyze the error probability between the actual information i encoded by C and the estimated information $\hat{i}$ after decoding the noisy string after the probing step.

**Figure 4:**
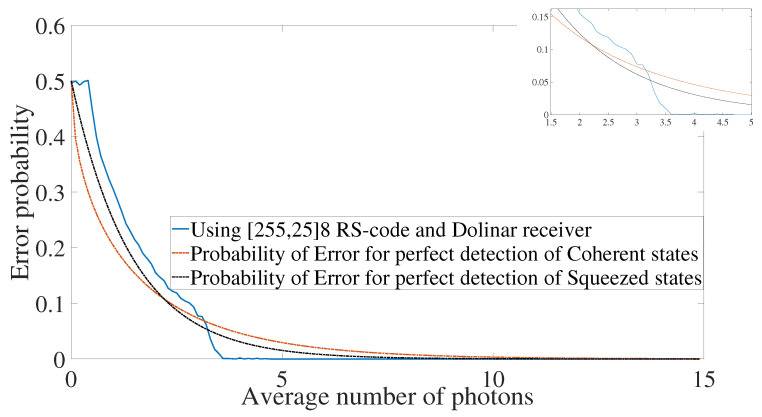
Error probability decay using ${\left[255,25\right]}_{8}$ Reed–Solomon code and Dolinar receiver for $\kappa_{0}=0.1$ and $\kappa_{1}=0.95$.

A sample plot is shown in Figure 4. For a fixed rate $R=\frac{K}{N}$, where *N* and *K* are the length and dimension of the classical code, respectively, we compute the error probability for several values of the average number of photons. The receiver is also fixed. For the particular case of Figure 4, we used a heterodyne receiver. The analysis described in detail for the heterodyne and Dolinar receivers below focuses on the crosspoint or threshold where using the classical code gives improvement compared to the optimal receiver using coherent or squeezed states. As an example, the value of the threshold using RS code with rate R=25/255 and Dolinar receiver is equal to 3.2, as one can see in Figure 4. Notice that the threshold depends on the slope of the error probability curve for each code and receiver. Therefore, it depends on the family of the code, the parameters of the code, and the receiver used. See below the error mitigation behavior analyzed under the perspective of the threshold for three families of classical codes, with several rates, and two types of receivers.

### 4.1. Heterodyne Receiver

For fixed values of transmissivities, $\kappa_{0}$ and $\kappa_{1}$, the analysis given in this paper consider the behavior of error mitigation for different codes. Because of analytical difficulties in deriving a closed formula for the error probability using classical codes, we adopted numerical simulations as the means of comparison between our case of study and the literature.

**Figure 5:**
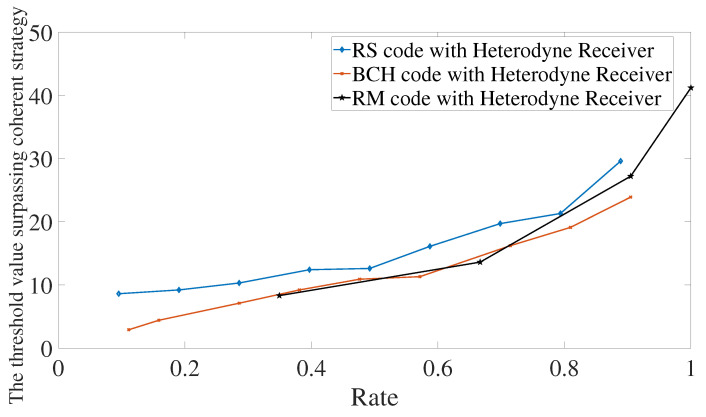
Error mitigation using Reed–Solomon, BCH, or Reed–Muller codes. All codes are considered in conjunction with a heterodyne receiver. The memory cells have transmissivities equal to $\kappa_{0}=0.1$ and $\kappa_{1}=0.95$.

The first characterization is presented in Figure 5. The behaviors of the three codes are similar. First of all, as the rate increases, it also increases the value of the threshold. Increasing the rate means that we have less redundancy and, therefore, the code has the error-correction capability decreased, which imposes the need for probing with higher energy in order to surpass the optimal strategies. Secondly, the threshold values go up to 45 photons. This means that we can keep in the quantum regime for surpassing the optimal strategies when using classical codes.

Comparing between codes, BCH codes and Reed–Muller codes have similar performances. However, it is important to remember that there are some limitations in using Reed–Muller codes since the choice of parameters does not have much freedom compared with BCH and Reed–Solomon codes. For Reed–Solomon codes, we see that they request probing with more energy, but such an increase of energy is less than 5 photons. Therefore, in practical applications, one would choose the code that has an encoder and decoder at the disposal with the lowest complexities.

**Figure 6:**
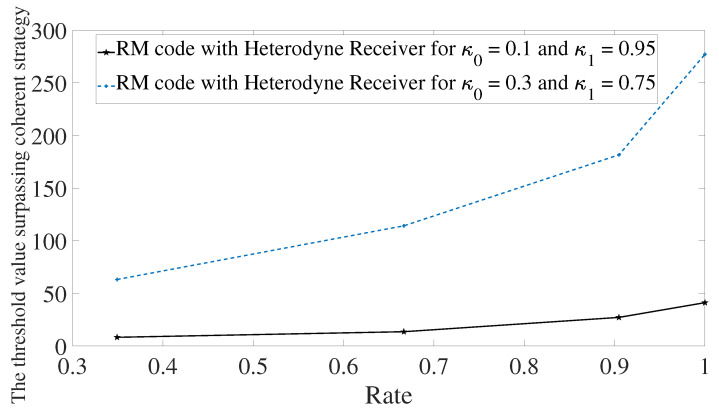
Comparison of performance using Reed–Muller codes for memory cells with different transmissivities. Solid line refers to memory cells with trasmittivities given by $\kappa_{0}=0.1$ and $\kappa_{1}=0.95$. Dotted line refers to memory cells with transmittivities $\kappa_{0}=0.3$ and $\kappa_{1}=0.75$.

Next, the behavior of the error mitigation is investigated for different values of transmissivities and using Reed–Muller codes. The first aspect one can see is that the performance highly depends on the difference between the transmissivities $\kappa_{0}$ and $\kappa_{1}$. For transmissivities where $\kappa_{0}$ and $\kappa_{1}$ are closer, the output probe states have position and momentum which are closer. Therefore, the heterodyne receiver in conjunction with maximum likelihood estimator has a higher probability of estimating the value encoded in the memory cell erroneously. This will imply in a receiver output string with more errors. For a code with the same rate to surpass the optimal receiver, it needs more probing energy so that error probability decreases. This is the reason why one needs more energy for $\kappa_{0}=0.3$ and $\kappa_{1}=0.75$, and why the slope of the curve is higher than the case with $\kappa_{0}=0.1$ and $\kappa_{1}=0.95$. See Figure 6. For Reed–Solomon and BCH codes, we have the same behavior.

### 4.2. Dolinar Receiver

The Dolinar receiver is a powerful receiver that can be used for achieving the Helstrom bound on the error probability of distinguishing quantum states. As mentioned before, it also comes with an inherited complexity due to its adaptive character. Nonetheless, it can be implemented in practice [20]. For the analysis shown below, we need to impose some defects on the Dolinar receiver, otherwise the results obtained would be unrealistic. The structure and procedure for implementing the Dolinar receiver is the one explained in Section 3.2.2. The defect imposed for the results below is over the efficiency of the photodetector, which we consider to be equal to 0.9. This value is close to the one obtained with the current technology for some photodetectors. One additional defect that could be added is dark counting. However, to consider an experiment with dark counting would need additional parameters, such as the rate of measurement over the memory cells. The complexity in dealing with these details could fade the importance of the codes used and mislead the analysis.

**Figure 7:**
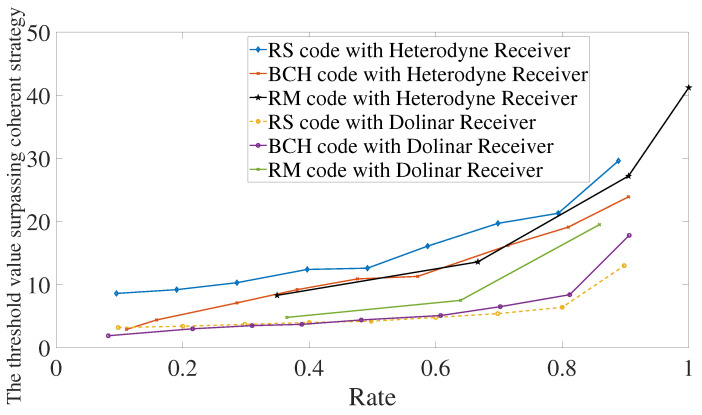
Comparison between heterodyne receiver and Dolinar receiver when using Reed–Solomon, BCH, and Reed–Muller codes. We are considering that the photodetector used in the Dolinar receiver has efficiency equal to 0.9 and memory cells have transmissivities $\kappa_{0}=0.1$ and $\kappa_{1}=0.95$.

**Figure 8:**
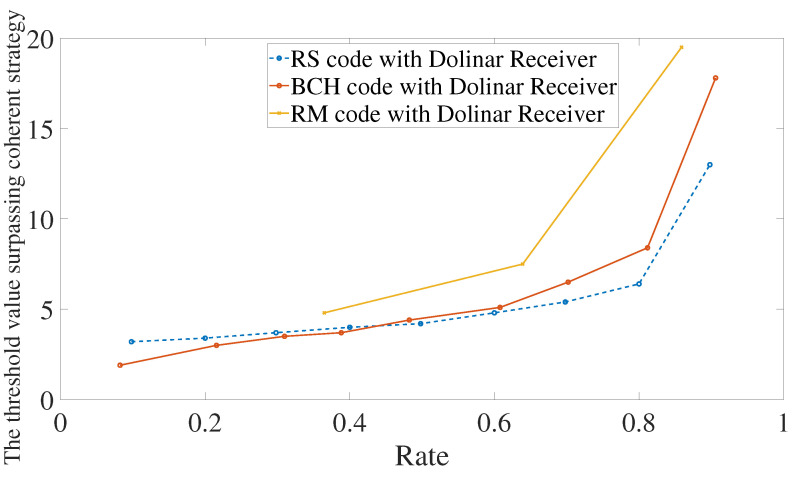
Error mitigation using Reed–Solomon, BCH, or Reed–Muller codes in conjunction with Dolinar receiver. We are considering that the photodetector used in the Dolinar receiver has efficiency equal to 0.9 and memory cells have transmissivities $\kappa_{0}=0.1$ and $\kappa_{1}=0.95$.

Let the reflexivities be $\kappa_{0}=0.1$ and $\kappa_{1}=0.95$. It is shown in Figure 7 the performance using Reed–Solomon, BCH, or Reed–Muller codes in conjunction with a heterodyne or Dolinar receiver. Reed–Solomon and BCH codes in conjunction with Dolinar receiver give the best performance between them all. For low rates, BCH codes with heterodyne or Dolinar receivers give similar results. In particular, for rates below 0.1, the threshold value of using BCH codes with the heterodyne receiver is 3.2 and with Dolinar receiver is 1.9. However, the difference between heterodyne and Dolinar receiver accentuates for a higher rate. The performance using the Dolinar receiver is shown in detail in Figure 8.

The performance of using Reed–Solomon or BCH codes in conjunction with Dolinar receiver is quite similar. For some rate R∈[0.4,0.5], Reed–Solomon codes show better error mitigation than BCH codes. However, for practical reasons, BCH codes may be the best choice. The reason is that Reed–Solomon codes are constructed in an extended field $F_{2^{s}}$, for some integer *s*, of $F_{2}$. This imposes the need to constantly decompose the coordinates of the codewords on a basis of $F_{2^{s}}$ over $F_{2}$. Such decomposition is implemented in the encoding and when writing the received string to feed the decoder. It is a not-so-demanding process, but may be substantial for choosing in favor of BCH codes.

There is a last point to consider. The accomplishments presented using Dolinar receiver could be due to the receiver and, therefore, no code is needed. However, this is not true. Since the efficiency of the photodetector is below one, we have that the error probability without code but using Dolinar receiver is bounded from below. In particular, for the efficiency of 0.9 considered in our simulation and for any value of the average number of photons, the error probability is always above 0.12%. So, to improve further the error mitigation, one needs to amend additional tools, such as classical codes.

## 5. Final Remarks

We have shown that classical error-correcting codes can be used as a tool to reduce error probability in quantum reading. They have been applied in a short length range. Even so, error mitigation is accomplished. Above all, they can exhibit improvements when compared with optimal strategies using coherent or two-mode squeezed states once a threshold is crossed. All of these for two types of receivers, heterodyne receiver or Dolinar receiver. We also studied the situation when the channel transmissivities are closer. It was shown that the error mitigation deteriorates but one can still surpass the optimal strategies probing with a state with higher energy. The same conclusion is obtained for the Dolinar receiver. As an overall conclusion of the analysis presented, BCH codes are the optimal code choice for error mitigation in the quantum reading task among the families of codes considered.

Several aspects could be further investigated in future works. Firstly, how the error probability mitigation behaves using codes with larger lengths and different rates. Some examples of possible choices of codes are LDPC and Turbo codes. It is expected that using codes with larger lengths and lower rates reduces the error probability. However, we do not know how this reduction will behave in this or in different scenarios. Secondly, analytical upper and lower bounds need to be obtained for a broader understanding of the current results. Lastly, it is expected that classical codes performance depends on the weight distribution of the code. This result may help to derive the error bounds mentioned before.

## Data Availability

Not applicable.
